# Incidence of statin use in older adults with and without cardiovascular disease and diabetes mellitus, January 2008- March 2018

**DOI:** 10.1371/journal.pone.0223515

**Published:** 2019-12-05

**Authors:** Catherine A. Panozzo, Lesley H. Curtis, James Marshall, Lawrence Fine, Barbara L. Wells, Jeffrey S. Brown, Kevin Haynes, Pamala A. Pawloski, Adrian F. Hernandez, Sarah Malek, Beth Syat, Richard Platt

**Affiliations:** 1 Harvard Pilgrim Health Care Institute and Harvard Medical School, Boston, MA, United States of America; 2 Department of Population Health Sciences, Duke University School of Medicine and Duke Clinical Research Institute, Durham, NC, United States of America; 3 Division of Cardiovascular Sciences, National Heart, Lung, and Blood Institute, Bethesda, MD, United States of America; 4 HealthCore, Inc., Wilmington, DE, United States of America; 5 HealthPartners Institute, Minneapolis, MD, United States of America; University of British Columbia, CANADA

## Abstract

**Background:**

Data from randomized controlled trials and observational studies on older adults who take statins for primary prevention of atherosclerotic cardiovascular disease are limited. To determine the incidence of statin use in older adults with and without cardiovascular disease (CVD) and/or diabetes (DM), we conducted a descriptive observational study.

**Methods:**

The cohort consisted of health plan members in the NIH Collaboratory Distributed Research Network aged >75 years who had continuous drug and medical benefits for ≥183 days during the study period, January 1, 2008- March 31, 2018. We defined DM and CVD using diagnosis codes, and identified statins using dispensing data. Statin use was considered incident if a member had no evidence of statin exposure in the claims during the previous 183 days, and the use was considered long-term if statins were supplied for ≥180 days. Incidence rates were reported among members with and without CVD and/or diabetes, and stratified by year, sex, and age group.

**Results:**

Among 757,569 eligible members, 109,306 older adults initiated statins and 54,624 became long-term users. Health plan members with CVD had the highest incidence of statin use (143.9 initiators per 1,000 member-years for CVD & DM; 114.5 initiators per 1,000 member-years for CVD & No DM). Among health plan members without CVD, those with DM had rates of statin use that were over two times higher than members without DM (76.1 versus 34.5 initiators per 1,000 member-years, respectively). Statin initiation remained steady throughout 2008–2016, was slightly higher in males, and declined with increasing age.

**Conclusion:**

Incidence of statin use varied by CVD and DM comorbidity, and was lowest among those without CVD. These results highlight the potential clinical equipoise to conduct large pragmatic clinical trials to generate evidence that could be used to inform future blood cholesterol guidelines.

## Introduction

Limited data from either randomized controlled trials or observational studies on older adults who take statins for primary prevention of atherosclerotic cardiovascular disease make it difficult to inform clinicians on decisions for statin use in this population [1, 2]. To begin to address this gap, investigators associated with the JUPITER (Justification for Use of Statins in Prevention: An Intervention Trial Evaluating Rosuvastatin) [3] and HOPE-3 (Heart Outcomes Prevention Evaluation) [4] primary prevention clinical trials conducted a meta-analysis of age-specific outcome data, and found a 26% relative risk reduction in nonfatal myocardial infarction, nonfatal stroke, or cardiovascular death among patients aged >70 years [5]. However, given the modest number of adults aged ≥80 years in these trials, and the continued uncertainties around questions related to hemorrhagic stroke, cognitive function, adherence, and quality of life, further study is warranted [5]. Results from the STAREE (Statin Therapy for Reducing Events in the Elderly) efficacy trial, when available, will help fill some of these knowledge gaps, but generalizability will remain a concern as only individuals without diabetes were included, the ethnic diversity of the population is limited, and it is not intending to evaluate a full range of patient-centered outcomes (NCT02099123) [1].

Considering the limited evidence available, the 2018 American Heart Association (AHA)/ American College of Cardiology (ACC) Guideline on the Treatment of Blood Cholesterol to Reduce Atherosclerotic Cardiovascular Risk in Adults recommends that physicians decide whether to initiate primary prevention statin therapy in patients >75 based on clinical assessment and risk discussion [6]. Given the uncertainty in the area, growing aging population in the US, and to determine the feasibility of conducting a future clinical trial, we conducted a descriptive observational study of statin use in older adults with and without cardiovascular disease (CVD) and diabetes mellitus (DM).

## Materials and methods

### Data sources

The NIH Collaboratory Distributed Research Network (DRN) consists of ten data partners, including four national health insurers, five regional health insurers, and one prospective, longitudinal study [7]. For this query, one national insurer (HealthCore, Inc.) and two regional insurers (HealthPartners and Harvard Pilgrim Health Care) totaling >60 million health plan members with data formatted to the Sentinel Common Data Model (SCDM) participated [8]. Data partner participation was voluntary and based on availability.

### Cohort

The cohort consisted of health plan members aged >75 who had continuous drug and medical insurance coverage for a minimum of 183 days with an allowance for gaps in coverage of ≤45 days. To be eligible for the cohort that assessed long-term statin use, within the query period, patients were required to have an additional 180 days of continuous enrollment adjacent to and after the other 183 day enrollment requirement. We stratified the cohort based on whether patients had CVD or DM diagnosed on or within 183-days of the index statin dispensing date (or simply during the 183-day enrollment requirement if the patient did not use statins) which resulted in four statin user patient groups, including those with: 1) CVD & DM; 2) CVD & No DM; 3) No CVD & DM; and 4) No CVD & No DM.

We defined DM and CVD using International Classification of Diseases, Ninth Revision, Clinical Modification (ICD-9-CM) code algorithms previously described in the literature, using a 183-day lookback period. Specifically, we defined DM with the ICD-9-CM code “250*,” requiring ≥1 code in the inpatient setting or ≥2 codes on separate days in the outpatient setting [9]. We defined CVD as having experienced any of the following three conditions: myocardial infarction (ICD-9-CM: 410*, 411*, 412*, 413*, or 414* in the principal position of an inpatient claim) [10], heart failure (ICD-9-CM: 428* in any care setting with ≥2 codes) [11], or stroke (ICD-9-CM: 430*, 431*, 432*, 433*, 434*, 435* in the principal position of an inpatient claim) [12, 13]. We mapped the validated ICD-9-CM algorithms to the International Classification of Diseases, Tenth Revision (ICD-10-CM) codes using the Centers for Medicare and Medicaid Services General Equivalence Mapping files [14, 15].

### Exposures

We identified statins dispensed in the outpatient setting using National Drug Codes. Statin use was considered incident if a patient had no evidence of statin exposure in the claims during the 183-day period before the index dispensing.

### Descriptive analyses

We used the Sentinel System Cohort Identification and Descriptive Analysis (CIDA) tool to conduct the analyses (S1 File) [16]. Each data partner executed an identical CIDA analytic package against their local data and then returned aggregate information to the NIH Collaboratory DRN Coordinating Center for compilation.

We reported the incidence rate of any duration of statin use overall and by DM and cardiovascular comorbidity group, calendar year among years with compete data (January 1, 2008- December 31, 2016), sex, and age group (76–80 years, 81–85 years, and ≥86 years). We also assessed the incidence rate of long-term statin use, based on a minimum of a 180-day supply of statins, allowing for gaps of ≤30 days and applying an exposure episode extension of 30 days. We chose a cut-point of 180 days to define long-term use because randomized controlled trials commonly follow patients for outcomes for a minimum of 6 months (~180 days) and we sought to provide information useful for planning pragmatic clinical trials [17]. Finally, we described summary statistics characterizing the length of statin exposure episodes, and explored reasons for why statin exposure episodes ended.

### Human subjects

We requested a waiver of informed consent and each participating organization received not human subjects IRB determination from their respective IRBs.

## Results

### Overall incidence and description of exposure episodes

**Fig 1:**
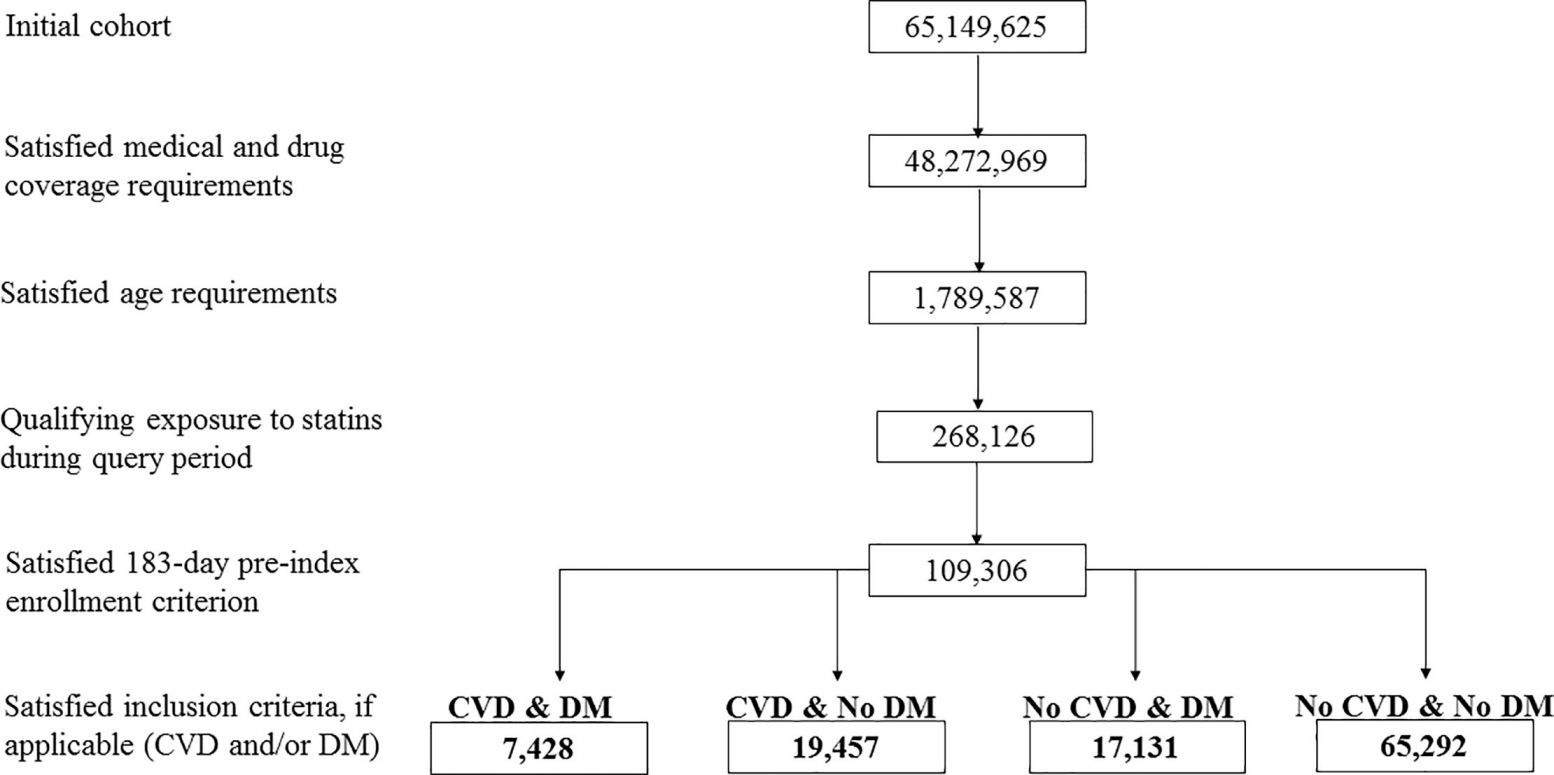
Cohort attrition for CVD & DM, CVD & No DM, No CVD & DM, and No CVD & No DM cohorts, all duration of statin use among initiators aged >75 years, NIH Collaboratory Distributed Research Network, January 1, 2008- March 31, 2018. Abbreviations: CVD = cardiovascular disease; DM = diabetes mellitus.

Among an eligible population of 757,569 members contributing 2,338,358 member years, 109,306 older adults initiated statins (incident rate [IR] = 46.7 statin initiators per 1,000 member-years) from January 1, 2008-March 31, 2018 (Fig 1, Table 1). We observed 54,624 long-term new users of statins (IR = 26.8 initiators per 1,000 member-years). Across all incident statin users, the median duration of statin use was 179 days (interquartile range [IQR], 90–499), and across members who initiated and used statins long-term, the median duration of statin use was 500 days (IQR, 300–931 days). Most statin episode ended due to discontinuation of therapy unrelated to health plan disenrollment, death, or end of observation period. Disenrollment and death combined accounted for no more than 35% of the observed exposure episode truncations in a given calendar year.

**Table 1:** Statin initiation among older adults aged >75 years with and without cardiovascular disease and diabetes mellitus, NIH Collaboratory Distributed Research Database, January 1, 2008- March 31, 2018.

						Summary Statistics: Length of Statin Episode in Days				
Level	New Users	Dispensings^a^	Eligible Members^b^	Eligible Member Years^b^	Incidence Rate per 1,000 Member-Years	Min	Q1	Median	Q3	Max
**Initiators with any duration of statin use**										
Overall	109,306	884,839	757,569	2,338,358	46.7	1	90	179	499	3,722
CVD & DM	7,428	56,915	57,279	51,618	143.9	1	60	154	431	3,650
CVD & No DM	19,457	172,004	183,345	169,925	114.5	1	78	188	509	3,649
No CVD & DM	17,131	131,702	136,667	225,157	76.1	1	90	167	484	3,719
No CVD & No DM	65,292	524,225	693,026	1,891,658	34.5	1	90	180	510	3,722
**Initiators with long-term statin use**										
Overall	54,624	794,587	696,397	2,034,702	26.8	180	300	500	931	3,722
CVD & DM	3,468	49,694	44,204	40,815	85.0	180	289	462	811	3,650
CVD & No DM	10,055	154,480	140,486	136,072	73.9	180	293	490	890	3,649
No CVD & DM	8,378	117,830	121,897	196,495	42.6	180	297	493	915	3,719
No CVD & No DM	32,724	472,588	635,668	1,661,321	19.7	180	300	509	965	3,722

Abbreviations: Min = minimum; Q1 = first quartile; Q3 = third quartile; Max = maximum; CVD = cardiovascular disease; DM = diabetes mellitus

^a^Dispensings are the total number of dispensings used to create episodes. Same day dispensings are counted as 1.

^b^Eligible Members and Eligible Member-Years reflect the number of patients who met all cohort entry criteria on at least one day during the query period

### Incidence by CVD and DM strata

Health plan members with CVD with and without co-morbid DM had the highest incidence of statin use (143.9 initiators per 1,000 member-years for CVD & DM; 114.5 initiators per 1,000 member-years for CVD & No DM). Among health plan members without CVD, those with DM had rates of statin use that were over two times higher than members without DM (76.1 versus 34.5 initiators per 1,000 member-years, respectively). Patterns of incident statin use among long-term users in each DM and CVD stratum were similar to the patterns observed across initiators with any duration of use, but the incidence rates were 35 to 44% lower in each stratum.

### Incidence by calendar year, sex, and age group

**Fig 2:**
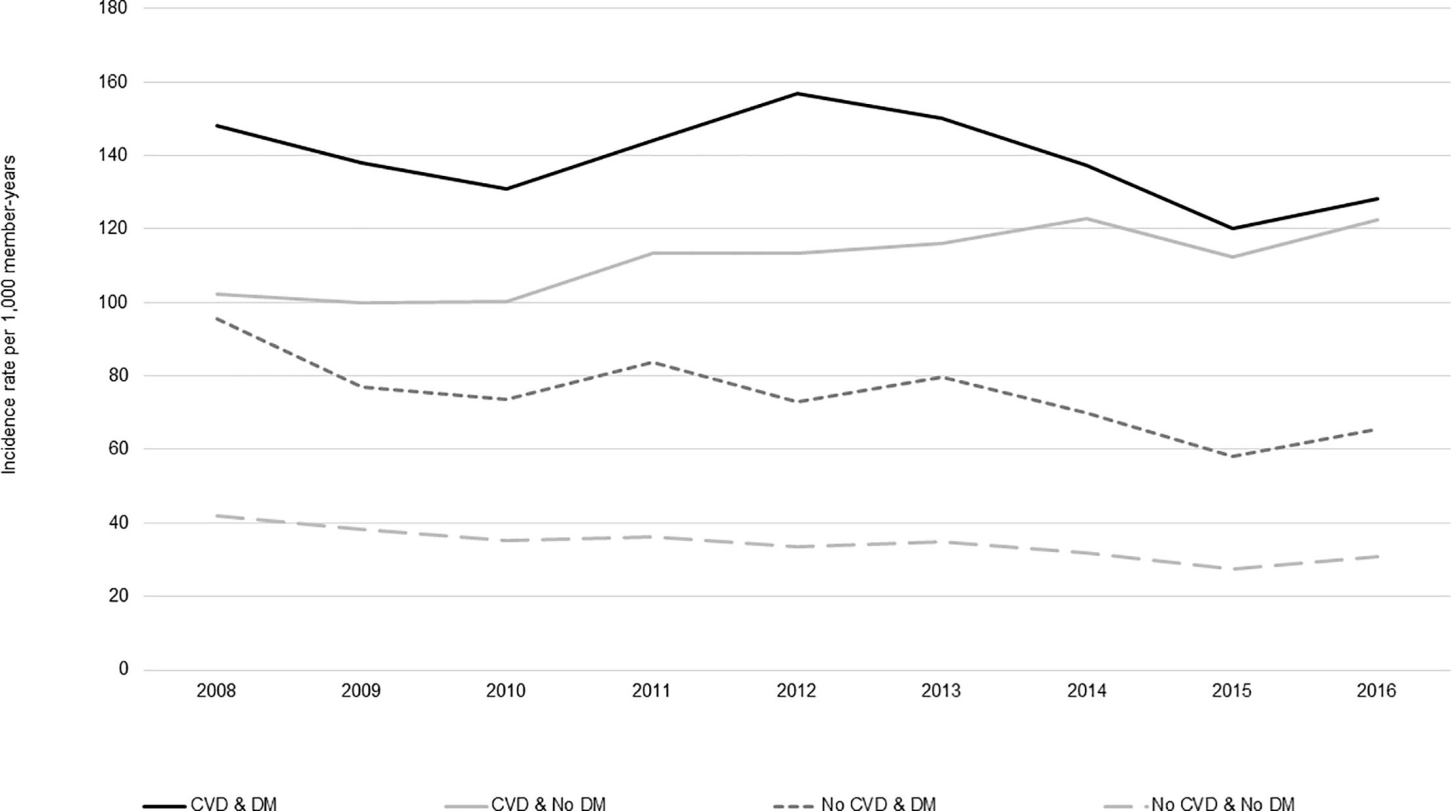
Statin initiation rates among older adults aged >75 years with and without cardiovascular disease and diabetes mellitus by calendar year, NIH Collaboratory Distributed Research Network, January 1, 2008- December 31, 2016*. Abbreviations: CVD = cardiovascular disease; DM = diabetes mellitus *Data were incomplete in 2017 and 2018 so these years were excluded from this analysis to prevent misleading information that can arise from unstable data.

**Fig 3:**
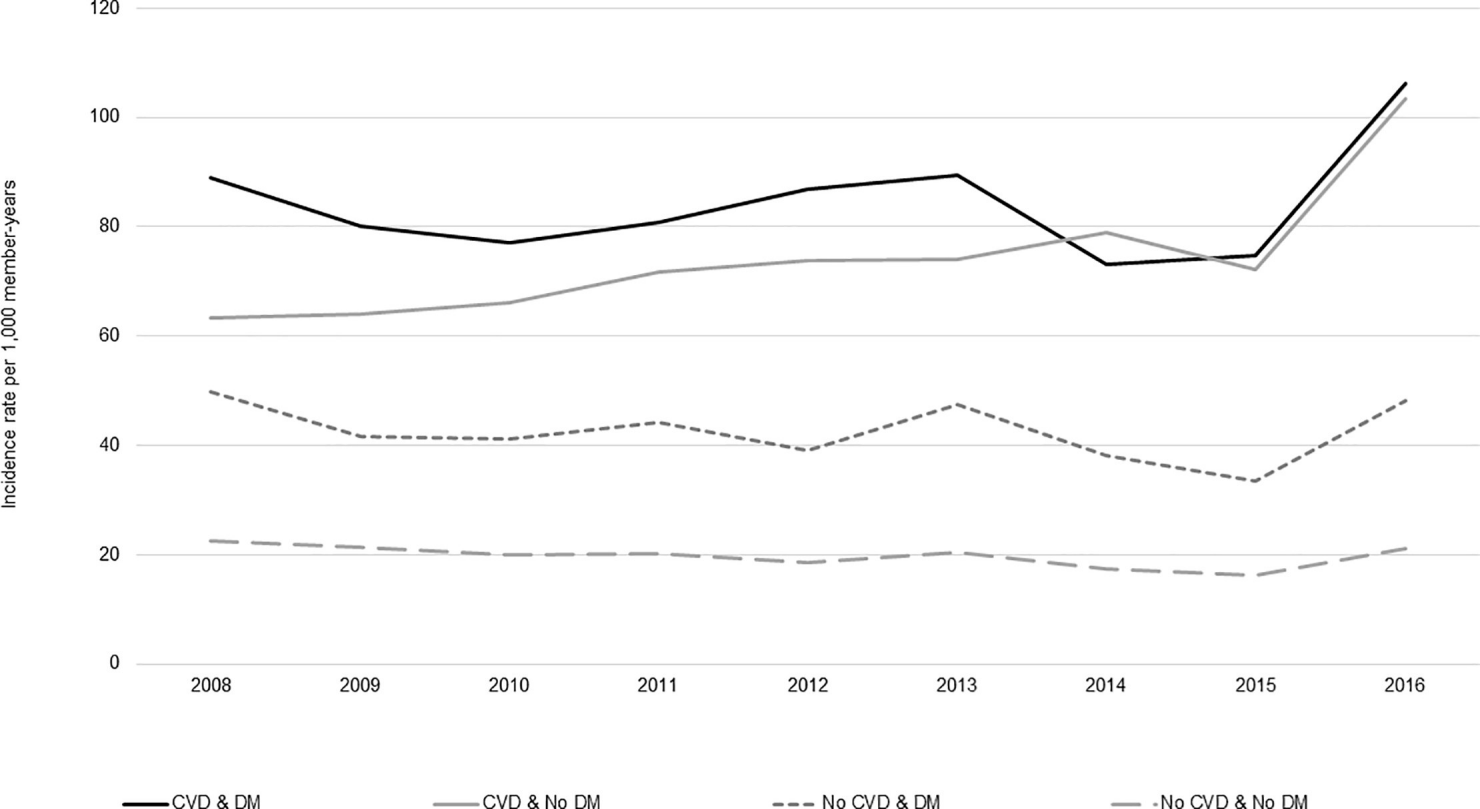
Statin initiation rates among older adults aged >75 years with and without cardiovascular disease and diabetes mellitus by calendar year, who go on to use statins long-term,* NIH Collaboratory Distributed Research Network, January 1, 2008- December 31, 2016†. Abbreviations: CVD = cardiovascular disease; DM = diabetes mellitus *Long-term use was defined as a minimum of 180 days of statin exposure based on the number of days supplied, allowing for gaps of up to 30 days and including an exposure episode extension period of 30 days ^†^Data were incomplete in 2017 and 2018 so these years were excluded from this analysis to prevent misleading information that can arise from unstable data.

**Fig 4:**
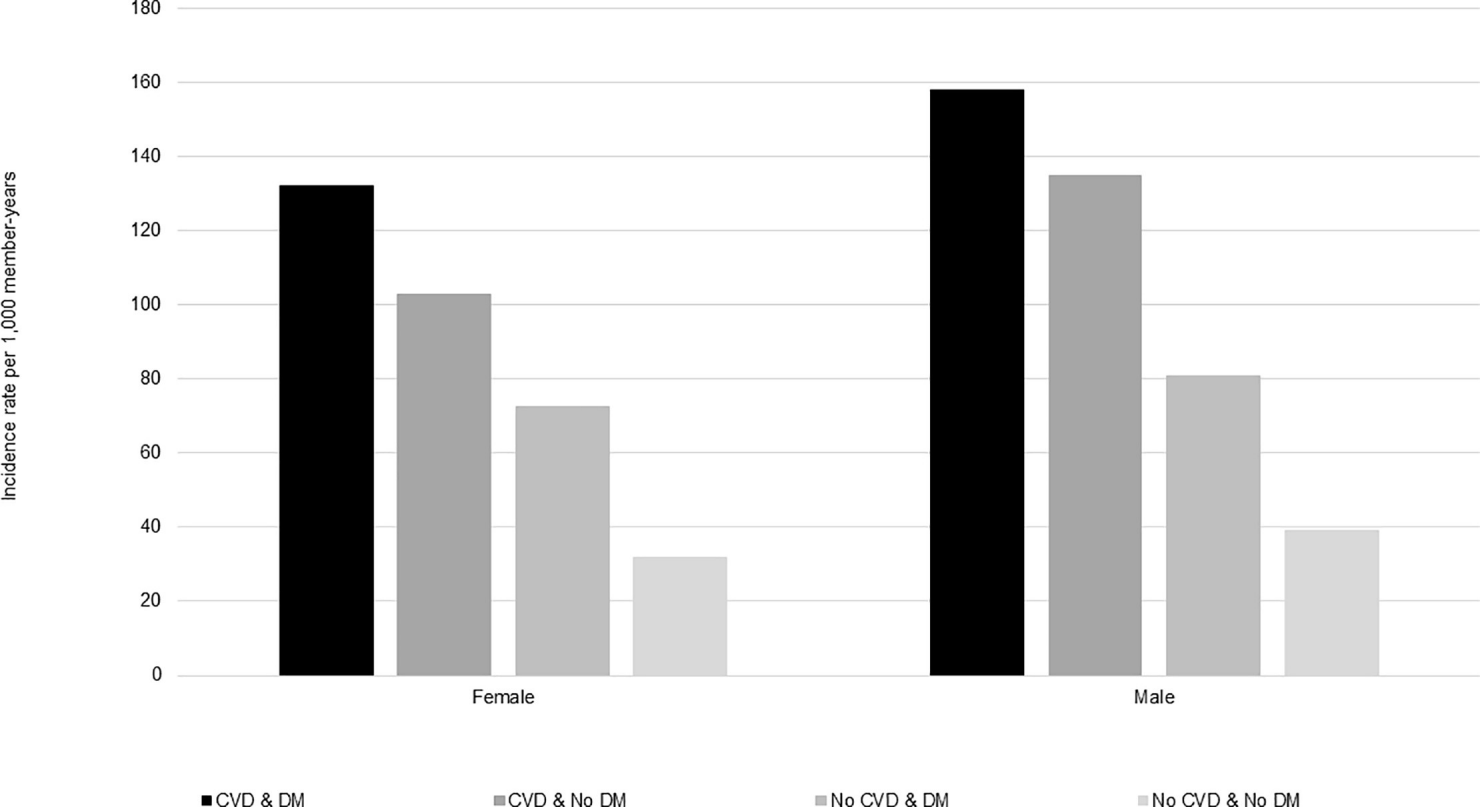
Statin initiation rates among older adults aged >75 years with and without cardiovascular disease and diabetes mellitus by sex, NIH Collaboratory Distributed Research Network, January 1, 2008- March 31, 2018. Abbreviations: CVD = cardiovascular disease; DM = diabetes mellitus.

**Fig 5:**
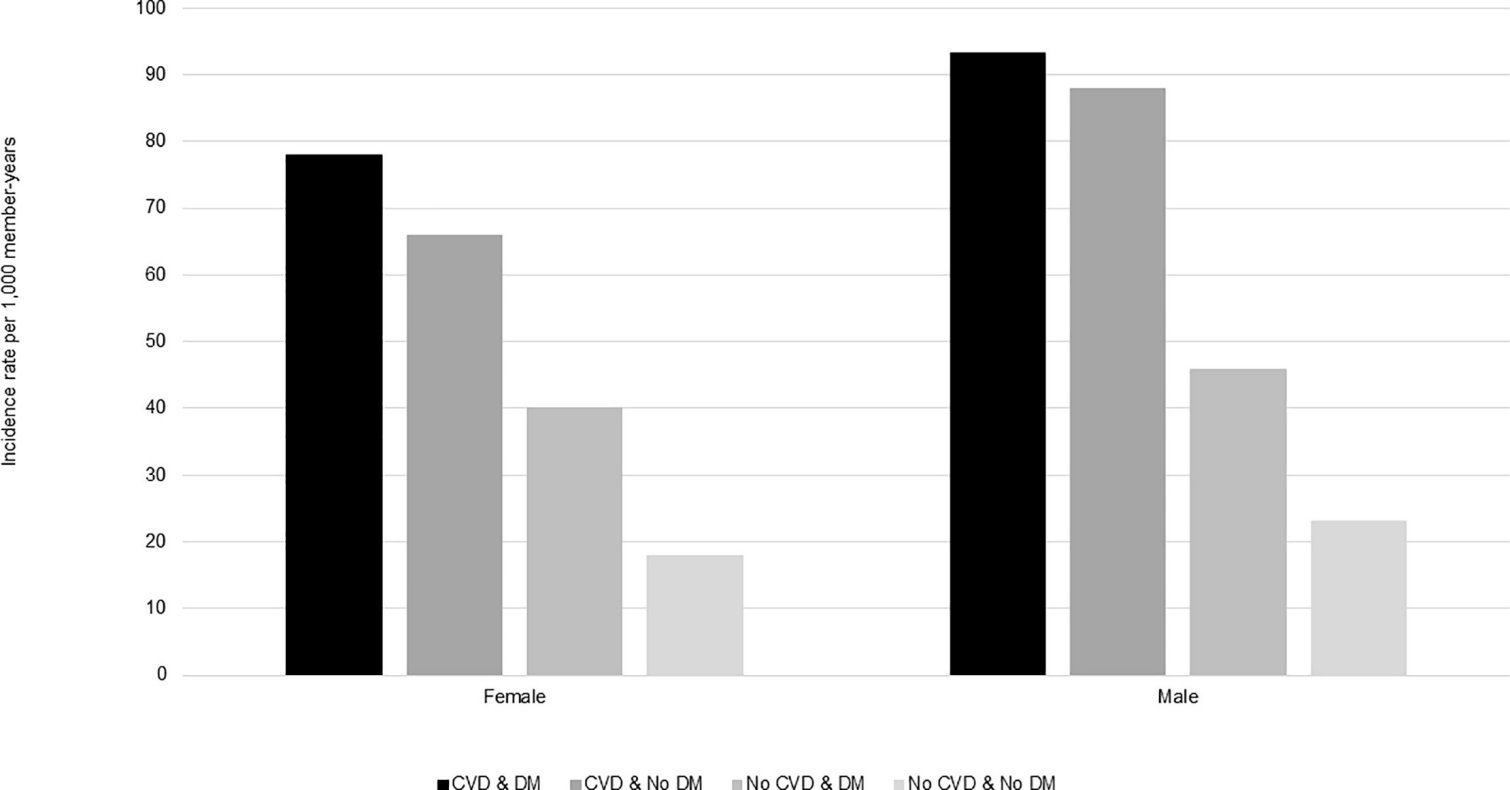
Statin initiation rates among older adults aged >75 years with and without cardiovascular disease and diabetes mellitus by sex, and who go on to use statins long-term,* NIH Collaboratory Distributed Research Network, January 1, 2008- March 31, 2018. Abbreviations: CVD = cardiovascular disease; DM = diabetes mellitus *Long-term use was defined as a minimum of 180 days of statin exposure based on the number of days supplied, allowing for gaps of up to 30 days and including an exposure episode extension period of 30 days.

**Fig 6:**
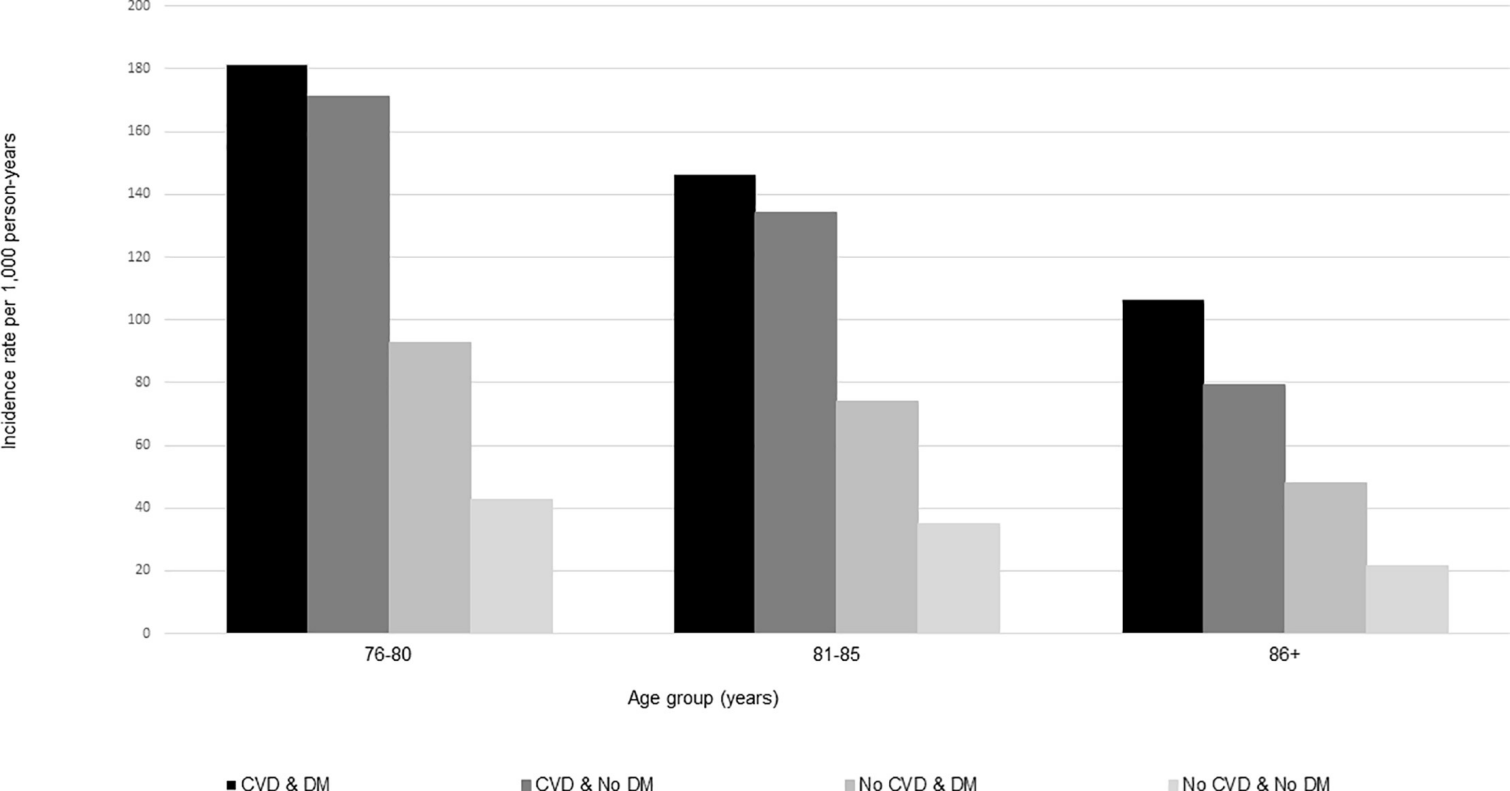
Statin initiation rates among older adults aged >75 years with and without cardiovascular disease and diabetes mellitus by age group, NIH Collaboratory Distributed Research Network, January 1, 2008- March 31, 2018. Abbreviations: CVD = cardiovascular disease; DM = diabetes mellitus.

**Fig 7:**
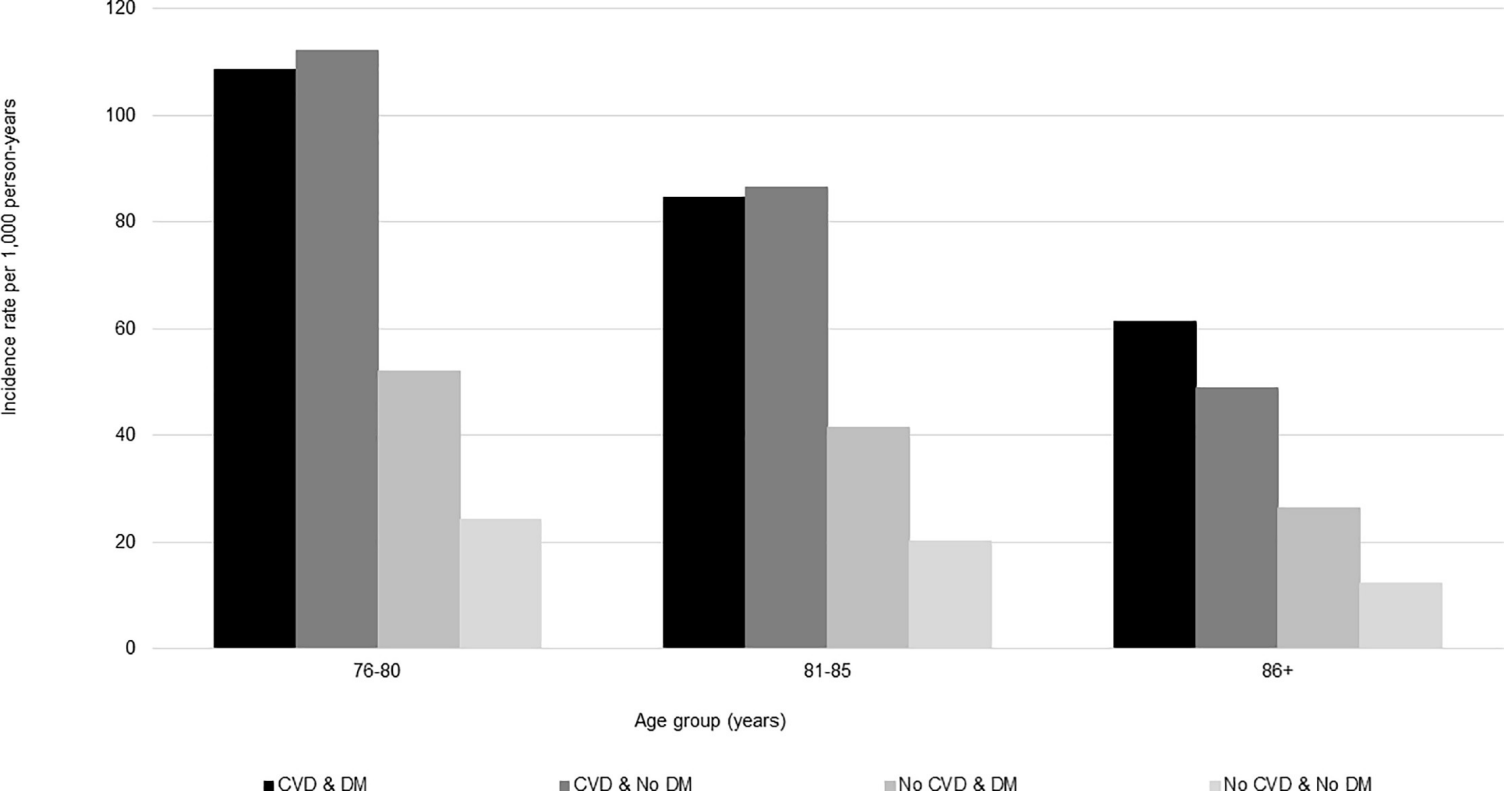
Statin initiation rates among older adults aged >75 years with and without cardiovascular disease and diabetes mellitus by age group, and who go on to use statins long-term,* NIH Collaboratory Distributed Research Network, January 1, 2008- March 31, 2018. Abbreviations: CVD = cardiovascular disease; DM = diabetes mellitus *Long-term use was defined as a minimum of 180 days of statin exposure based on the number of days supplied, allowing for gaps of up to 30 days and including an exposure episode extension period of 30 days.

Across the study years for which data were complete (2008–2016), incidence of statin use was steady (Fig 2); however, the incidence of long-term users increased from 2015–2016, especially among patients with CVD (Fig 3). Compared to females, males had slightly higher rates of statin use across all CVD and DM cohorts (Figs 4 and 5). Incident use of statins declined with age; in all four study cohorts, those aged 76–80 had the highest incidence, followed by those aged 81–85, and those aged ≥86 (Figs 6 and 7).

## Discussion

This study reports the incidence of statin use in a large cohort of older adults in the US with and without CVD and DM over a long study period (>10 years). Relatively low rates of statin initiation among older adults, especially among patients taking statins for primary prevention, highlight the potential clinical equipoise to conduct large pragmatic clinical trials to generate precise, real-world statin effectiveness and safety estimates that could be used to inform future blood cholesterol guidelines. While low incidence of statin use does not necessarily translate to low prevalence of statin use in older adults, most well-designed effectiveness and safety studies would require studying incident users so we focused on these patients.

Incidence of statin use varied by the presence of CVD and DM comorbidities. Consistent with other epidemiologic studies or guidelines, patients with CVD had the highest rates of statin use [6, 18], and patients with DM had initiation rates that were twice as high as patients without diabetes [6, 19]. Moreover, patients with both underlying conditions (CVD & DM) initiated statins at four times the rate of those with neither of the conditions (No CVD & No DM). Although some studies suggest an association between statin use and new onset DM, since our population focused on incident users of statins, this association would not explain why patients with DM have higher rates of statin use. Rather, we suspect that the AHA/ACA blood cholesterol guidelines that emphasize patients with DM as an important high-risk group drive the prescribing patterns observed in this study [6, 19–21]. The incidence of statins was slightly higher in males versus females and highest among the youngest age group (i.e., 76–80 years), but despite the 2013 AHA/ACC blood cholesterol guideline [22, 23], trends were consistent from 2008–2016. Another study conducted using the United Kingdom’s (U.K.) The Health Improvement Network primary care database estimated statin initiation with prescription data from 1995–2013, and similarly found that rates of statin initiation declined with increasing age in older adults, from a maximum of 51.01 per 1,000 person-years among adults aged 75–84 years in 2006 to 23.45 per 1,000 person years among adults aged ≥85 years in 2006 [24]. This study also found that males initiated statins at a higher rate than females beginning in 2004, but all adults aged ≥18 years contributed data to sex-specific analyses [24].

Even after ruling out health plan disenrollment and death as reasons for discontinuation, our study found that approximately 50% of older adults who initiate statins do not use statins long-term, defined in our study as at least six months (180 days) Medical literature of other older adult populations supports our findings, suggesting that many older patients who initiate statins may receive little to no benefit due to premature discontinuation [25, 26]. For example, one cohort study using linked population-based administrative claims data from Canada found that among adults aged >65 years, 40.1% of patients with acute coronary syndrome, 36.1% of patients with coronary artery disease, and 25.4% of patients taking stains for primary prevention remained adherent to their statin medication two-years following their initial prescription (25). A systematic review analyzing >3 million statin initiators aged ≥65 years identified poor adherence after one-year of follow-up, with just 62.3% of secondary prevention users and 47.9% of primary prevention users reporting adherence to statins [26]. In a study using a 10% random sample of younger adults (mean age, 55.9 years; standard deviation 10.3 years) in the IMS LifeLink Health Plan Claims Database from July 1997- December 2008, patients’ adherence levels to statins also tended to decline over time [27], although perhaps not as much as studies focused exclusively on older adult populations. For example, among the most adherent group, defined as having a proportion of days covered by a statin prescription of ≥0.80 in their first year, 70% of adults remained at this level in year 2 [27]. Our study found that approximately half of all statin initiators discontinued statin use by six months, and that having diabetes may have modified the length of adherence in the primary and secondary prevention cohorts (i.e., No CVD & DM had a shorter median follow-up time than the No CVD & No DM (167 vs. 180 days); similarly, CVD & DM had a shorter median follow-up time than CVD & No DM (154 vs. 188 days). Shorter durations of statin use among patients with DM could be due to physician or patient awareness that statins may worsen (or cause) diabetes [20]. Other studies have shown that discontinuation of statins is also frequent in younger populations with and without diabetes [28, 29], and one recent study in older adults found that diabetes, anxiety, and increasing age may predict discontinuation [30]. Further investigation to understand the reasons and variation for poor long-term adherence would be helpful in designing targeted interventions that could improve adherence to statins.

This study had several strengths. First, we accessed data from >60 million health plan members. The large size of the data network enabled us to stratify our cohort of older adults by various demographic and underlying conditions that were considered important to NIH but would have been impossible to do using population-based survey such as the National Health and Nutrition Examination Survey [31]. Second, we used highly curated data formatted to the Sentinel Common Data Model and pre-existing Sentinel System analytic tools which allowed for timely and efficient analysis across a large population in a distributed network. Finally, statin use was measured by outpatient pharmacy dispensings rather than prescriptions or self-report.

This study was also subject to some limitations. First, although the source population was large, it was not necessarily representative of the general population of older adults, as it only included those enrolled in commercial insurance most frequently covered by either employers or Medicare Advantage plans. While the percentage of Medicare beneficiaries enrolling in a Medicare Advantage plan increased from 9.7% in 2008 to 20.4% in 2018 [32], Medicare Advantage plans continue to comprise a much smaller proportion of the Medicare beneficiary population than Medicare fee-for-service plans, and characteristics of who enrolls in Medicare Advantage versus Medicare fee-for-service plans may vary. For example, from 2006–2009, Medicare Advantage beneficiaries were less likely to self-report a chronic condition than those enrolling in Medicare fee-for-service plans [33]. Second, as we only had access to administrative claims data, we could not define CVD and DM with clinical or laboratory values or conduct medical chart review. Third, while we explored general reasons for truncation of statin exposure episodes, we did not explore potential associations between discontinuation and toxicity (e.g., rhabdomyolysis) and other non-administrative reasons for discontinuing statins in detail due to the nature of claims data. However, while we note that discontinuation by or before 6 months (180 days) did not seem to vary by sex (Figs 4 and 5) or age group (Figs 6 and 7), the overall median duration of statin use was lower among patients with diabetes compared to those without diabetes (Table 1) Finally, given that we only characterized the duration of the first statin exposure episode, we cannot comment on the proportion who may have discontinued statin use only temporarily. A previous study conducted in the Boston area suggests that many patients, even those who discontinue statin use due to statin-related events, later re-initiate statins [29]; however, this study was conducted in a younger population (mean age 61.1 years, standard deviation 13.1). Nonetheless, additional implications include that our population of “incident” users may actually consist of a mix of older adults who are either 1) true initiators or 2) re-initiators of statins following a medication lapse extending beyond the washout that was used to define incidence (i.e., 183 days).

In conclusion, this descriptive study found relatively low rates of statin initiation among older adults and that statin initiation varied by CVD and DM status. The results from this study highlight the potential clinical equipoise to conduct large pragmatic clinical trials to generate evidence that could be used to inform future blood cholesterol guidelines.

## Supporting information

S1 File SAS analytic program for NIH collaboratory distributed research network statin query. (SAS)
